# Lymph Node Involvement in Advanced Gastric Cancer in the Era of Multimodal Treatment—Oncological and Surgical Perspective

**DOI:** 10.3390/cancers13102509

**Published:** 2021-05-20

**Authors:** Zuzanna Pelc, Magdalena Skórzewska, Karol Rawicz-Pruszyński, Wojciech P. Polkowski

**Affiliations:** Department of Surgical Oncology, Medical University of Lublin, Radziwiłłowska 13 St, 20-080 Lublin, Poland; zuzanna.torun@gmail.com (Z.P.); magdalenaskorzewska@umlub.pl (M.S.); wojciech.polkowski@umlub.pl (W.P.P.)

**Keywords:** advanced gastric cancer, neoadjuvant chemotherapy, lymph node metastases

## Abstract

**Simple Summary:**

Gastric cancer (GC) continues to be one of the major oncological challenges on a global scale. The role of neoadjuvant chemotherapy (NAC) in GC is to downstage primary tumour, eliminate potential micrometastases, and increase the chance for radical resection. Although systemic treatment prolongs the survival in advanced GC, persistent lymph node (LN) metastases indicate poor prognosis. Therefore, further identification of prognostic factors after NAC is urgent and could positively influence clinical outcomes. This article aimed to review the actual trends and future perspectives in multimodal therapy of advanced GC, with a particular interest in the post-neoadjuvant pathological nodal stage. Since downstaged and primarily node-negative patients show a similar prognosis, the main target for NAC in advanced GC should be nodal clearance. Adequate staging and personalised perioperative therapy seem to be of great importance in the multimodal treatment of GC.

**Abstract:**

Gastric cancer (GC) continues to be one of the major oncological challenges on a global scale. The role of neoadjuvant chemotherapy (NAC) in GC is to downstage primary tumour, eliminate potential micrometastases, and increase the chance for radical resection. Although systemic treatment prolongs the survival in advanced GC, persistent lymph node (LN) metastases indicate poor prognosis. Further identification of prognostic factors after NAC is urgent and could positively influence clinical outcomes. This article aimed to review the actual trends and future perspectives in multimodal therapy of advanced GC, with a particular interest in the post-neoadjuvant pathological nodal stage. A favourable prognostic impact for ypN0 patients is observed, either due to truly negative LN before the start of therapy or because preoperative therapy achieved a pathologically complete nodal response. Ongoing trials investigating the extent of lymphadenectomy after neoadjuvant therapy will standardise the LN dissection from the multimodal therapy perspective. Since downstaged and primarily node-negative patients show a similar prognosis, the main target for NAC in advanced GC should be nodal clearance. Adequate staging and personalised perioperative therapy seem to be of great importance in the multimodal treatment of GC.

## 1. Introduction

Gastric cancer (GC) continues to be one of the major oncological challenges on a global scale. According to GLOBOCAN 2020 data, GC remains the fifth most common cancer and the third most deadly neoplasm causing nearly 769,000 deaths in 2020 [1]. Curative management in early GC patients reaches nearly 90%. Unfortunately, lymph node (LN) metastases significantly decrease the 5-year overall survival (OS) to 70–80% in stage N1/N2, and to 30% in stage N3 [2,3]. A 5-years OS in advanced GC patients treated with optimal multimodal therapy, based on systemic chemotherapy and surgery does not exceed 38%, as shown in FNCLCC and FFCD Multicenter Phase III Trial [4]. However, the role of neoadjuvant chemotherapy (NAC) in nodal metastasis remains unknown and constituted the aim of JCOG trials [5,6]

Currently, perioperative and adjuvant chemotherapy (CTH) is indicated in advanced GC (stage IB-III) [7]. The role of neoadjuvant chemotherapy (NAC) is to downstage primary tumour, eliminate potential micrometastases, and increase the chance for radical resection [1,8,9]. Pathologic response to NAC is treated as an independent predictor of OS and 3-year disease-specific survival [10,11,12]. Although systemic treatment prolongs the survival in advanced GC, persistent LN metastases indicates poor prognosis. Therefore, further identification of prognostic factors after NAC is urgent and could positively influence clinical outcomes [2]. The American Joint Committee on Cancer (AJCC) in its 8th edition created a separate staging system for GC patients who underwent preoperative therapy [13]. Post-neoadjuvant pathological stage (yp) is considered as important survival predictor [14]. It allowed distinguishing several groups of patients by nodal involvement: cN0/ypN0 (node-negative), cN+/ypN0 (downstaged N0) and ypN+ (node-positive). Indeed, one of the most valid prognostic factors in advanced GC is lymph node (LN) involvement [1,15,16]. Despite multimodal treatment, the prognosis for ypN+ patients is poor, suggesting further insight into the treatment strategy. Recognition of occult LN metastases in a preoperative setting remains a challenge, since preoperative independent predictive factors produce area under the receiver operating characteristics curve of only 0.660 [17]. Thus surgeons need to be aware of limitations in preoperative prediction of the LN metastasis. Efficient verification of LN status is essential considering the clinical issues of undertreatment and overtreatment.

This article aimed to review the actual trends and future perspectives in multimodal therapy of advanced GC, with particular interest on post-neoadjuvant pathological nodal stage.

## 2. Preoperative Verification of LN Status

Verification of LN is essential while determining the treatment pathway, followed by extent of lymphadenectomy during gastrectomy. The role of appropriate preoperative assessment is crucial in the era of multidisciplinary and multimodal treatment. In order to establish a new golden standard for nodal staging, several ongoing trials aim to assess imaging characteristics for nodal staging [18] (NCT04028375), compare radiological techniques with histopathological verification [19] (NCT04440605), and determine the sensitivity of sentinel lymph node sampling [20] (NCT03049345).

### 2.1. Computed Tomography

Computed tomography (CT) is widely recognized as the primary method for staging and detecting distant metastases, determining surgical treatment [21]. Accuracy of preoperative cN staging is strongly dependent on cT stage and histological type of GC [22]. In early GC, LN metastases are detected with low sensitivity of only 34% [23]. The principal criterion of LN invasion in CT is nodal diameter, whereas other include: circular shape, heterogeneous enhancement, necrosis or decline of fatty LN hilum [24]. However, undefined cutoff values represent one of the primary limitations. Many radiologists performing multidetector CT arbitrarily accept a threshold of 6 mm for the celiac axis LN’s diameter and 8 mm for perigastric LN [25]. A German study showed that even 55% of cancerous-infiltrated LN remain smaller than 5 mm [26], which implies high possibility of false-negative and false-positive results. Limitations are related to poor visualisation of nodal engagement [21,27], nodal location dependency and sensitivity rate of 44.4% [22]. Moreover, in up to 30% of negative CT scans, endoscopic ultrasound (EUS) reveals nodal involvement [27,28]. Another restriction is associated with ambiguous restaging before potential gastrectomy. According to Gertsen et al., restaging CT prevents only 1% of unnecessary laparotomies, although preoperative assessment was performed before completing NAC [29]. High volume centres present contrasting numbers with negative prognostic value (NPV) of LN involvement at a level of 90.1% among patients with early GC [21,30]. However, in advanced GC Kagedan et al. estimated NPV of LN metastases at 43.3%, emphasizing the issue of cN understaging [31]. On the other hand, Yamamoto et al. investigated a correlation between the histological type of GC and accuracy of preoperative LN assessment: well-differentiated GC patients had more frequent overdiagnosis of LN involvement than undifferentiated GC (50% vs. 13.3%) [22].

### 2.2. Endoscopic Ultrasonography (EUS)

The effectiveness of nodal staging in EUS varies from 30% to 90% [10]. Perigastric LN suspected of metastasis occur as enlarged, round, hypoechoic and homogeneous structures. Unfortunately, lymphadenitis provides similar clinical image. An effective manner to distinguish those two processes is fine-needle aspiration (FNA) for cytological assessment, which should be performed unless the primary tumour and large vessels are in close proximity [10]. Another limitation results from suboptimal visualisation of regional LNs and poor quality of restaging, particularly after chemoradiotherapy [32,33]. Moreover, Ikoma et al. suggested that EUS could be eliminated from routinely clinical staging due to the low predictive value of cN assessment for patients undergoing NAC [17]. Extensive differences in sensitivity (16.7–96.8%) and specificity (48.4–100.0%) classify EUS as unreliable in detecting and excluding LN metastases [25]. However, according to recent data, the role of EUS in clinical staging of advanced GC should be maintained, underlying its correlation with further patient’s prognosis [33].

### 2.3. Magnetic Resonance Imaging

In terms of N staging, Magnetic Resonance Imaging (MRI) remains inferior to CT or EUS [34]. Restraints arise from costs and time of examination [18]. Sensitivity and specificity rates reach 85% and 67%, respectively and remain steady over time [35,36]. Additionally, there is no standardised protocol for defining LN metastases [37]. However, the role of MRI in nodal verification may change—diffusion-weighted (DW) MRI establishes LN involvement not by diameter but based on the integrity of cell membranes and tissue consistency [18]. Nevertheless, MRI is currently not recommended for nodal assessment [10].

### 2.4. Fluorodeoxyglucose Positron Emission Tomography

Fluorodeoxyglucose positron emission tomography (FDG PET) is not a universal method in GC imaging since up to 30% of non-FDG avid primary tumour (particularly mucinous type) [38]. Its sensitivity and specificity in the detection of LN metastases have been reported to be 41–51% and 86–100%, respectively [39]. FDG PET sensitivity rates, in particular, are lower compared to CT and EUS (56% vs. 78% and 50% vs. 73%, respectively) [40]. However, PET compiled with CT (PET-CT) could be valuable in detecting occult metastases helping to avoid redundant gastrectomy [41]. Fluorodeoxythymidine (F-FLT) PET is limited in its ability for detecting LN metastasis due to insufficient spatial resolution of PET component [42]. The primary use of PET-CT in clinical trials refers to an assessment of metabolic response after NAC identifying patients who do not benefit from a preoperative setting (e.g., cN+ units) and should be referred to surgery or undergo treatment modification [32,43]. Currently, the clinical usefulness of FDG PET after NAC in locally advanced GC is limited [44]. MUNICON trial was the first one proving the role of PET in verifying initial response to NAC and underlying prognostic rather than predictive role [45]. Since no firm conclusions can be made on solely performing FDG-PET/CT [46], the results of the PLASTIC study should be awaited [47], which hypothesized that performing PET and staging laparoscopy(SL) for locally advanced GC results in a change of treatment strategy in 27% of patients.

### 2.5. Staging Laparoscopy

SL with lavage cytology is a valuable staging procedure to tailor the treatment strategy and avoid unnecessary laparotomy in advanced GC [48]. Despite great value of SL in peritoneal metastases assessment [49,50], it has a potential disadvantage for N staging, since it cannot provide complete exploration of the regional lymph nodes [51]. On the other hand, direct visualization and laparoscopic ultrasound (LUS) of loco-regional LNs may allow removal of an entire LN for an extensive pathological examination [52].Presence of bulky regional lymph nodes in diagnostic imaging suggest a high potential for peritoneal spread, which should be considered as an additional indication for SL [53,54]. In case of suspected extra-regional LN metastases (stage IV) additional biopsies can be performed [54].

The comparison of selected imaging techniques for nodal staging in advanced GC is shown in Table 1.

## 3. Nodal Status from an Oncological Perspective

### Neoadjuvant Chemotherapy

NAC is used in patients with localised and resectable GC and aims to allow radical resection and reduce possible microscopic spread. Its efficacy was proved in two flagship trials: MAGIC and FNCLCC. Based on the ECF scheme (epirubicin, cisplatin, and 5-fluorouracil), patients with stage II-III GC recorded an increase in 5-year SR and less advanced nodal disease when compared to surgery alone [61]. FNCLCC and FFCD Multicenter Phase III Trial verified the concept of perioperative 5-fluorouracil and cisplatin doublet revealing improvement of 5-y OS (24% vs. 38%) and irrelevant decrease in LN invasion [4].

MAGIC and FNCLCC studies set the stage for the multimodal treatment of advanced GC in the West. However, the golden standard, superior to ECF/ECX regimen, remains FLOT scheme (5-fluorouracil, folinic acid, oxaliplatin, and docetaxel) after publishing the clinically meaningful results of the AIO-FLOT trial [62]. Importantly, Al-Batran et al. achieved higher rate of ypN0 patients in the FLOT group (49% vs. 41%, *p* = 0.025), although despite this encouraging progress, cure rates oscillating at 40% are still insufficient.

Therefore new studies are being conducted, such as GASTRODOC Regimen (docetaxel, oxaliplatin and capecitabine), where Monti et al. veryfied whether four cycles of NAC were superior to perioperative CTH for locally advanced GC [63], providing higher progression-free survival (PFS) and nodal response rates (from 22% of cN0 to 34% of ypN0) in experimental group.

Management of signet ring GC remains a great challange due to its poor response to NAC. The unsatisfying outcomes result from higher prevalence of nodal disease at initial stage of treatment and lack of improvement in LN downstaging after NAC [64]. However, the French PRODIGE 19 trial comparing adjuvant CTH vs. perioperative CTH for resectable signet ring GC revealed higher OS and DFS in the perioperative CTH group [65]. Similar conclusions were introduced in the FLOT4-AIO study confirming greater effectiveness of the FLOT-4 regimen vs. ECF/ECX in histopathological regression of GC [66,67]. The outcomes from II-III ADCI 002 trial are awaited and may provide new insight for NAC efficacy and its influence for cN+ status in signet ring GC [65].

## 4. Immunotherapy

Targeted management is required for human epidermal receptor 2 (HER-2) positive GC patients. The addition of trastuzumab to NAC induces better OS and improves ypN stage comparing to CTH alone [68,69]. German phase II PETRARCA trial analysed standard FLOT regimen vs. FLOT with the addition of trastuzumab and pertuzumab in perioperative management [70]. The study additionally revealed higher nodal response and complete pathological response (pCR) in patients treated with CTH with immunotherapy. Whether the anti-HER-2 dual blockade is superior to trastuzumab alone will be revealed after the publication of the results of the INNOVATION trial [69]. Limitations of anti-HER-2 therapy include frequent dose modification due to leucopenia or diarrhea [70] and the necessity to reevaluate HER2 status after completion of neoadjuvant treatment.

Phase III KEYNOTE-585 trial assessed value of adding pembrolizumab to perioperative CTH in GC patients [71]. The study included patients with localised GC or esophageal-gastric junction (EGJ) adenocarcinoma and additionally evaluated pathological nodal response. The appliance of immunochemotherapy and achieving pathologic complete response is also analysed in the ICONIC trial (avelumab with FLOT regimen for operable GC) [72].

Patients with microsatellite instability (MSI) may benefit from nivolumab therapy due to more common PD-L1 expression [73], frequently present in patients with LN metastases. Typically, MSI+ GC is characterised by different biology, unfavourable outcome and is classified as prognostic biomarker in GC [74], indicating higher 5-years OS and DFS. MSI+ GC shows no clinical benefit from both NAC and adjuvant CTH. However, there is a possible gain in usage of immune checkpoint inhibitors (ICI), such as nivolumab or pembrolizumab. Partial regression of LN metastases was observed in a Japanese case report after 23 courses of nivolumab [75]. Currently, MSI testing is advocated for newly diagnosed GC patients regardless of the clinical stage [10,32]. The results of clinical trials show benefits from adding biological agents to the currently recommended perioperative treatment [67]. However, the results of EORTC-1707 VESTIGE trial119 are awaited. The research investigates the role of adjuvant immunotherapy with nivolumab and ipilimumab in patients with higher risk of relapse(e.g., ypN+) [76]. 

## 5. Status of Radio- and Radiochemo-Therapy in Perioperative Setting

Radiochemo-therapy (RCTH) in a preoperative setting clinically impacts tumour regression grade, and increases the chance for radical resection. Nevertheless, LN status remains the most valid prognostic factor of survival after neoadjuvant therapy and gastrectomy. The addition of RT by its Abscopal effect [34,77] results in less adequate detection of LN in histopathological examination [34].

The results of CROSS trial had a significant impact on OS in patients with esophageal and EGJ cancer who underwent neoadjuvant RCTH followed by radical surgical treatment [78]. Australian TOPGEAR trial investigates preoperative RCTH (MAGIC regimen) for patients with locally advanced GC [79,80]. Sada et al. in a retrospective cohort study aimed to verify the role of preoperative RT in GC treatment in patients with clinically positive LN [81]. More than one-third of cN+ GC showed pathologic nodal response with preoperative treatment. It was concluded that RT is associated with a higher response than CTH. However, patients with ypN+ disease had worse survival, regardless of whether they received adjuvant therapy. Analysis of data from the National Cancer Database (NCDB) outlined similar conclusions: nodal regression after NAC was 30% compared to 47% after neoadjuvant RCTH [82].

In the context of ypN+ patients, the role of adjuvant RCTH is limited. ARTIST trial verified the effect of RCTH versus CTH alone among GC patients after D2 gastrectomy [83]. Early results proved statistically insignificant improvement in 3-years DFS. Noteworthy, RCTH resulted in improved DFS when compared to CTH alone in subgroup of ypN+ patients (77.5% vs. 72.3%). 7 years later ARTIST-2 trial systematised these results, showing no advantage in RCTH, apart from ypN+ patients maintaining DFS advantage [84].

Successively, the Dutch study CRITICS-II compared perioperative CTH with preoperative CTH and postoperative RCTH in patients with resectable GC [85]. Postoperative RCTH did not improve OS compared to postoperative CTH. Additionally, poor postoperative patients’ compliance in both treatment groups was found, implicating a need for optimising preoperative treatment strategies.

## 6. Outcome of Neoadjuvant Therapy on LN Status

### 6.1. cN0/ypN0—Natural N0

Despite historically known inaccuracy of preoperative evaluation of cN status [86], The proportion of cN0 patients increases due to more sensitive diagnostic procedures and earlier identification of GC [87]. Natural N0 patients are reported with a similar prognosis as those with cN+/ypN0 [86], indicating ypN0 status is an important hallmark representing a successful preoperative treatment of GC regardless of pre-treatment cN status. However, primarily N0 groups are correlated with lower cT category than downstaged patients (60.1% cT3–4 vs. 76.8% cT3–4) [82]. On the other hand, ypN0 patients had markedly better overall survival than did ypN+ patients regardless of ypT status [86].Unfortunately, even 26% of primarily cN0 patients are pN+, further known as occult metastases [22]. Moreover, 41% of cases diagnosed as ypN0 reveal micrometastases, resulting in higher risk of GC recurrence [87]. LN micrometastases may remain unidentified during typical H+E staining. However, immunohistochemical technique based on cytokeratin evaluation is a successful method of LN micrometastases detection [88]. In the 7th edition of TNM classification LN micrometastases are classified as N+, although isolated tumour cells present in LN are categorized as N0 [89].

### 6.2. cN+/ypN0—Downstaged N0

In the MAGIC trial, patients with good response to NAC (ypN0) who underwent R0 resection had most favourable outcome in the entire cohort. The median OS in that subgroup of patients was not reached, since it was longer than the predicted censoring period. Brazilian research group proved that cN0 and cN+/ypN0 patients have similar prognosis [81]. Surprisingly, LN regression impacted DFS and decreased the risk of death more significantly than downstaging the primary tumour. Similar results were obtained by the Japanese research group showing no difference in 5-year OS between cN0 and cN+/ypN0 (72% vs. 69%) [86]. Moreover, the addition of anti-HER-2 agents to perioperative treatment resulted in a higher rate of ypN0 status when compared to CTH alone (68% vs. 39%) [70]. According to Stark et al., neoadjuvant RCTH caused more frequent nodal downstaging simultaneously with inferior survival benefit when compared to NAC alone [82]. Therefore, negative LN status is the most promising independent favourable prognostic factor for patients with GC.

### 6.3. ypN+—Node-Positive

There are discrepancies between cN+ GC patients treatment in the East and the West. In Asia, cN+ status implies the necessity of surgical assessment followed by adjuvant chemotherapy [90]. In the West, perioperative approach is recommended. Interestingly, the concept of multimodal treatment is gaining recognition also in the United States, where the Asian guidelines have been respected so far [91].

In ypN+ patients high risk of recurrence is observed, regardless of ypT status. This issue was addressed in the MAGIC trial. Patients who did not achieve pathological response after the perioperative treatment had poor prognosis. Furthermore, the adjuvant CTH compliance rate was 22.9%. Thus it was unclear whether the favourable outcome of perioperative treatment was achieved through adjuvant CTH or NAC alone [32] The impact of postoperative CTH among ypN+ patients shows no survival benefit [40]. Sada et al. suggested that cN+/ypN+ patients should be treated with second-line regimen or additional radiotherapy [81].

## 7. Nodal Status from the Surgical Perspective

Since LN involvement is one of the most important prognostic factors in advanced GC, technique and extent of lymphadenectomy is of crucial importance from surgical prespective [92]. The extent of LN dissection depends on primary tumour location and size, together with substantial risk factors for LN metastases: lymphovascular invasion, submucosal infiltration, poor differentiation, large tumour size and ulceration [7,10,93]. D2 lymphadenectomy is generally accepted as standard procedure during curative gastrectomy. While in the East it has been performed with satisfying outcomes for decades [93], in the West a consensus was reached relatively lately, indicating superiority of the D2 procedure [7,10]. It requires LN removal from the perigastric area (N1) along with the lymph nodes at the coeliac axis and its branches (N2). The benefit of an extended (D2) LN dissection is based upon four pillars: reliable staging with increased number of resected nodes; removal of potential LN metastases; risk reduction of loco-regional recurrence, and improvement of overall survival, albeit not shown in any of the previous randomised trials [92,94,95]. However, D2 lymphadenectomy may improve disease-specific survival in advanced GC with LN metastases, as recently shown by Italian trial [96].

**Figure 1:**
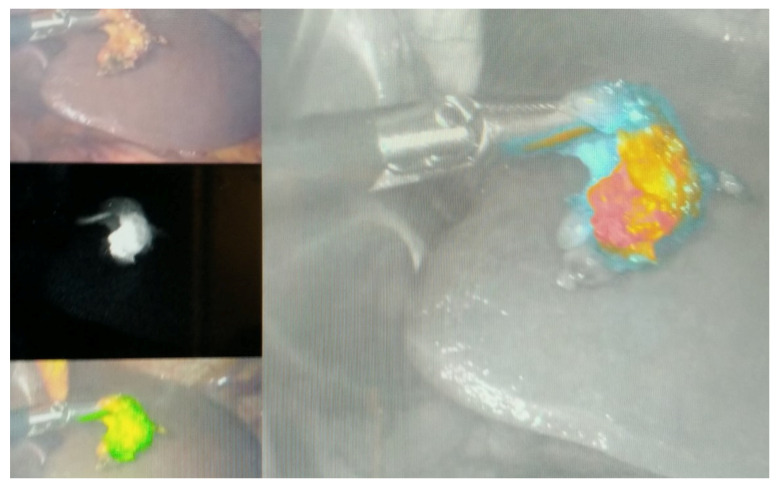
Fluorescence-guided LN dissection in GC patients (Department of Surgical Oncology, Medical University of Lublin).

If the number of harvested LNs is not adequate (less than 15 LNs pathologically examined), the proportion of positive LNs to the total LN harvest, called the LN ratio (LNR), is valuable prognostic factor [97]. A meta-analysis of 27 studies confirmed LNR as an independent prognostic factor in GC patients undergoing upfront surgery, where higher LNR was significantly related to poor long-term outcomes [98]. In patients treated with multimodal therapy, tumour diameter, Laurén intestinal subtype, no pathological tumour response, serosal infiltration and distant metastases were significantly associated with higher ypLNR [99]. More extensive (>D2) lymphadenectomy is suggested only in the experimental setting [100] or during gastrectomy with extra-regional LN metastases [93]. Ongoing trials investigating the extent of lymphadenectomy after neoadjuvant therapy will allow to standardize the LN dissection from the multimodal therapy perspective (NCT02139605, NCT03961373). Fluorescence-guided lymphadenectomy is a new, feasible technique which allows to detect LN metastases within fluorescent LN stations [101,102] (Figure 1). Indocyanine green can improve the LN harvest and reduce LN noncompliance without increased complications in patients undergoing D2 lymphadenectomy [103].

## 8. East vs. West Perspective

The differences in incidence and survival between Eastern and Western GC patients result from the disease’s biological characteristics and different screening and treatment strategy [104]. With an extended lymphadenectomy, more commonly performed in the East, more LNs are retrieved with a higher chance of detecting a positive node, resulting in a stage migration phenomenon [90]. However, the differences in surgical practice for GC between the East and the West have lessened and become standardized [105]. A recent study on the impact of the introduction of formal D2 lymphadenectomy in a Western setting resulted in improved LN sampling, decreased postoperative complications and improved survival of patients undergoing surgery for GC [106]. However, a wide variation remains in the multimodal treatment concept. In contrast to the perioperative approach in the West, adjuvant chemotherapy with S1 or XELOX regimen are used [104]. The collaboration between the East and the West will allow for a better understanding of the specific subtypes of GC and will facilitate future studies to improve treatment strategies [91].

## 9. Nodal Regression Grade

Histologic downstage of cN+ status results in prolonged survival [107,108]. Moreover, regression of LN status is more significant than regression of the primary tumour in predicting GC recurrence [107]. Additionally, LN regression, regardless of the percent of tumour cells in the primary site, independently influences the survival in patients who underwent NAC [109]. Factors increasing chances for higher nodal regression grade are lack of venous, lymphatic and perineural invasion, lower primary tumour depth and diameter, regardless of its location. Nodal regression grade might be applied as one of the endpoints in clinical trials verifying the efficacy of neoadjuvant treatment. Unnecessary reporting of nodal regression grade due to the higher prognostic value of LN’s residual tumour assessment in predicting the prognosis after NAC was highlighted in a study from the East [108]. Despite usage of similar classification of regressive changes, this result stays in contradiction with European findings [110]. Both, nodal response and the quality of that response correlates with long-term survival. A favourable prognostic impact for ypN0 patients is observed, either due to truly negative LN before the start of therapy or because preoperative therapy achieved a pathologically complete nodal response [111].

## 10. Conclusions

Since downstaged and primarily node-negative patients show similar prognosis, the main target for NAC in advanced GC should be the nodal clearance. More adequate staging and personalized perioperative therapy seem to be of great importance in the multimodal treatment of GC.

## Figures and Tables

**Table 1:** Comparison of selected imaging techniques for nodal staging in advanced GC.

Imaging Technique	Sensitivity	Specificity	PPV	NPV	Accuracy
CT	44.4 [22]–92% [21]	78.8 [35]–93.4% [22]	62.8–83.1% [21]	80.00% [22]	43.3 [31]–90.1% [21]
MRI	86% [36]	67% [36]	78% [55]	64% [56]	77.8% [56]
PET	40.3–61% [21]	97.7% [35]	69.9 [57]–85.8% [58]	95.2% [57]	46.1% [57]
EUS	63–83% [21]	80–95% [21]	75% [21]	98% [59]	94.1% [59]
SL	53% [60]	91% [60]	88% [60]	53% [60]	90% [60]

CT—computed tomography, MRI—magnetic resonance imaging, PET—positron emission tomography, EUS—endoscopic ultrasonography, SL—staging laparoscopy, PPV—positive predictive value; NPV—negative predictive value.

## Data Availability

Not applicable.
